# Secondary Cutaneous Involvement in Follicular Diffuse Lymphoma Treated with Helical Tomotherapy

**DOI:** 10.7759/cureus.1471

**Published:** 2017-07-14

**Authors:** A. Rashid Dar, Kevin Jordan, Slav Yartsev

**Affiliations:** 1 Department of Radiation Oncology, London Regional Cancer Program, Western University; 2 Physics, London Regional Cancer Program, Western University; 3 Physics, London Regional Cancer Program, Western University

**Keywords:** helical tomotherapy, cutaneous lymphoma

## Abstract

Non-Hodgkin’s lymphoma is a complex heterogeneous group of disease entities that involves nodal and extranodal tissues. Cutaneous involvement can occur either as a primary or secondary in course of disease. Radiation therapy with either total body or localized treatments is often used for local control and symptom relief, depending on the target volume. We describe a 60-year-old male with a remote history of stage IA left neck follicular lymphoma treated with radiation 20 years ago and previous relapses aggressively treated by chemotherapy. Treatment to a large volume of back and posterior shoulders on a helical tomotherapy radiotherapy system is reported. The skin lesions responded completely with no toxicity. Palliative radiotherapy to a fairly large and complex volume of skin with modest dose avoiding underlying critical tissues on tomotherapy is feasible, well tolerated with an excellent durable response, without compromising future chemotherapy and stem cell transplant for systemic relapse.

## Introduction

Non-Hodgkin’s lymphoma, a common haematological malignancy, occurs worldwide. Over the last two decades, a considerable progress has been made in classification and therapies of lymphomas [1]. While the primary cutaneous involvement of lymphoma is well reported [2-3], there is a paucity of reports about secondary involvement [4]. Palliation and local radiation therapy option for symptom relief and local control remain the standard of care using electron beam therapies [5]. However, these therapies experience several technical and dosimetric issues, especially for targets with irregular volume and large surface, as well as meeting the constraint for the organs at risk.

Tomotherapy technique use for scalp lesions is reported including our own [6], but there are only three tomotherapy reports in advanced lymphoma/leukemia with an excellent outcome: total body skin irradiation replacing electron beam [7-8] and the circumference of thorax [9]. We present the tomotherapy use for the large volume of the posterior back above the waist, posterior shoulders, and lower neck without compromising future chemotherapy and stem cell transplant. Informed consent statement was obtained for this study.

## Case presentation

A 60-year-old male, with no high-risk features presented with multiple asymptomatic skin lesions over upper back, posterior shoulders and anterior-lateral aspects of the skull. Biopsies had revealed follicular B-cell lymphoma, CD10, B-cell lymphoma (Bcl-2), Bcl-6 positive and negative for CD5, CD43. Twenty years ago, he received 35 Gy in 20 fractions curative radiotherapy to symptomatic masses in left neck stage I follicular lymphoma and stayed in remission for 17 years. Then he relapsed and received 25 Gy in 20 fractions to abdominal nodes with partial response and 25 Gy in 10 fractions to the nodular lesion on right temple of the scalp with complete response. On follow-up, he developed a mass over uvula. Biopsy showed diffuse large cell transformed lymphoma, CD20, CD10, Bcl-2, Bcl-6 positive and CD5 negative. The patient received Cyclophosphamide-Hydroxyldaunorubicin (DOXOrubicin)-ONCOVIN® (VinCRIStine)-Prednisone-riTUXimab (CHOP+R) eight cycles and one cycle of Rituximab, responded well with moderate side effects, remained in remission for the next 1.5 years when multiple new spots on the back of chest and shoulders became symptomatic. The lesions increased in number and appeared varying in size with no ulcerations. The biopsies confirmed low grade to grade three marker positive lesions, some nodules were infiltrating deeper, the superficial epidermis was clear. The patient received CCNU (Lomustine)-Etoposide-Prednisone-riTUXimab (CEP+R) three cycles with no response. Photodynamic therapy with 5-aminolevulinic acid topical application showed only a minimal response. Electron beam therapy was rejected due to dosimetric and technical constraints. Tomotherapy was chosen due to previous radiotherapy and recent aggressive chemotherapy and potential of future chemotherapy and transplant. There were a complete durable response and no toxicity, other than moderate erythema and pigmentation. Blood work also remained normal. Two years later he became symptomatic with positron emission tomography (PET) highly positive nodes treated with Gemcitabine-Dexamethasone-PLATINOL® (CISplatin)-Rituximab (GDP+R) combination three cycles, followed by standard auto stem cell transplant. The patient now, 24 years since original diagnosis and 1.5 years since transplant, remains well in clinical remission and with good quality of life. Skin lesions remain in complete remission four years since tomotherapy. Appropriate consent for publication was obtained from photographs.

**Figure 1:**
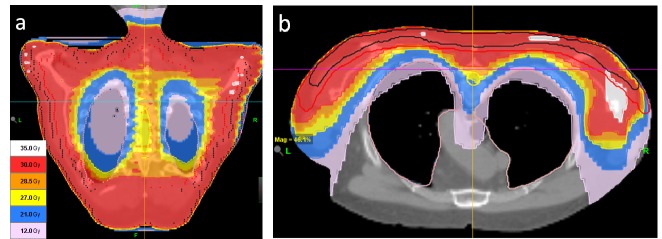
Planned dose distribution (a) coronal and (b) transversal cross sections

Helical tomotherapy was chosen over conventional multiple adjoining electron beams due to a large planning target volume (2,671 cm3), underlying critical tissues (lungs, spinal cord), and our experience with treatment of superficial sites with this technology [6]. The patient was positioned prone in VacLock (Merit Medical Systems, Inc, South Jordan, Utah) with forehead rest, arms extended, and quietly breathing and scanned on a 16-slice Phillips Big Bore computer tomography ( Philips Medical Systems, Cleveland, Ohio, United States) unit with a 3 mm inter-slice spacing. We recommend reviewing soft tissue windows on planning computed tomography (CT) to include deeper extensions of these nodular lesions to avoid any miss and normal tissue constraints. The clinical target volume was expanded 1 cm isotropically and touching the skin to create modified planning target volume (PTV). The PTV had an overlap with the target of previous radiation treatment in the area from cervical vertebrae (C4) to thoracic vertebra (T2). The biologically effective dose (BED) of 40 Gy was conservatively estimated for the previous treatment of 24.8 Gy to the spinal cord without considering repair during 20 years, leaving a room for up to 30 Gy in 15 fractions to the spinal cord within the PTV. The largest possible (5 cm) fan beam width was chosen to minimize the treatment time and a structure was added anteriorly to serve as a directional block for better sparing of the lungs and heart. The following parameters were used on tomotherapy planning station (version 4.2): a pitch of 0.287, an initial modulation factor of 2.5, dose-volume objectives of D65% = 2.5 Gy, D35% = 5 Gy, D25% = 10 Gy for the lungs, Dmax = 25 Gy for the cord, Dmax, Dmin, and D95% = 30 Gy for the PTV. The resulting planned dose distribution is shown in Figure 1. 

**Figure 2:**
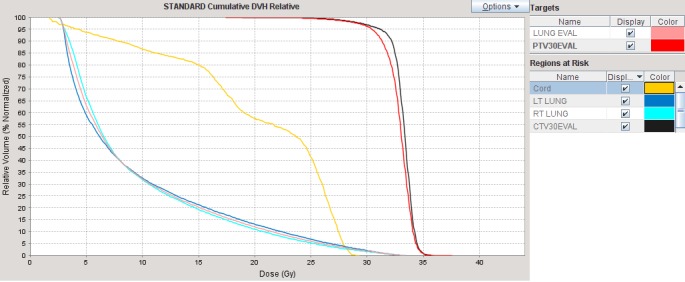
Dose-volume histogram

Prescription dose to the PTV was 30 Gy in 15 fractions to achieve a competing response. The planned skin dose was 95%-98% of the prescription, so no bolus was used. The lungs and spinal cord dose were well within tolerance: Figure 2.

**Figure 3:**
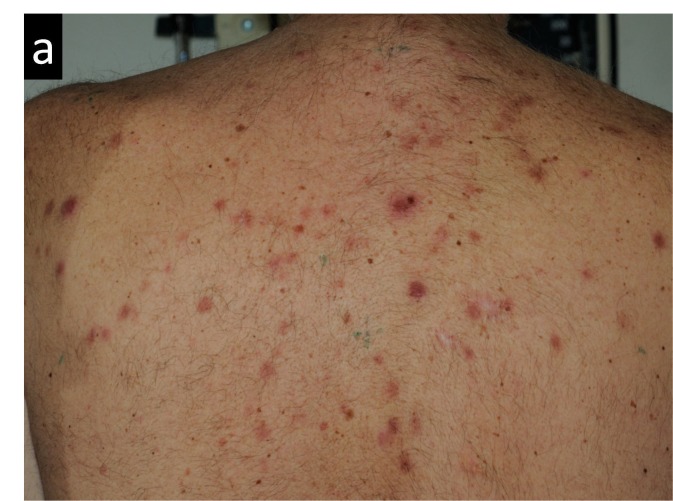
Radiation-induced changes in the patient’s back appearance after two fractions of radiation therapy

**Figure 4:**
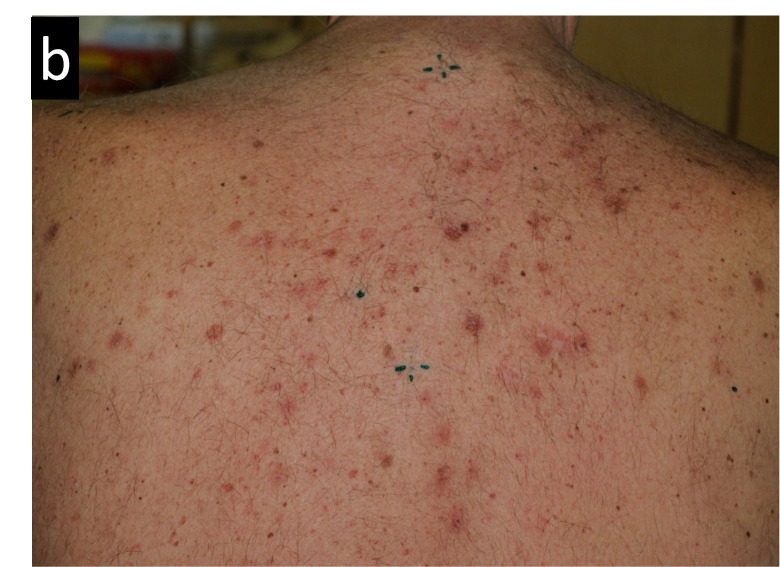
Radiation-induced changes in the patient’s back appearance after 14 fractions of radiation therapy

**Figure 5:**
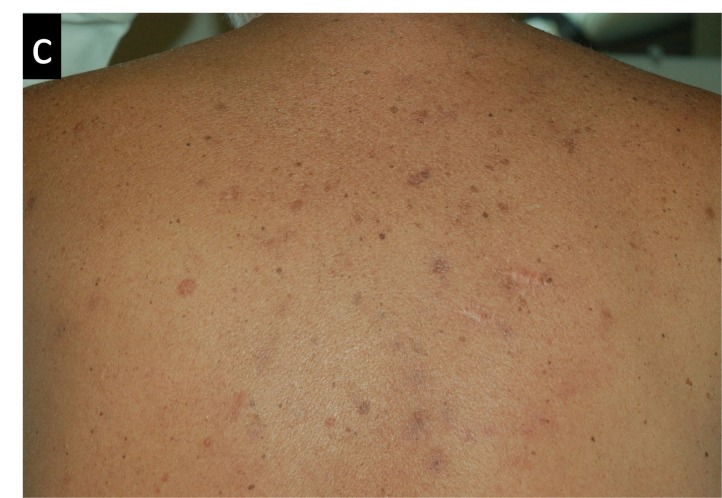
Radiation-induced changes in the patient’s back appearance three months after radiation therapy

**Figure 6:**
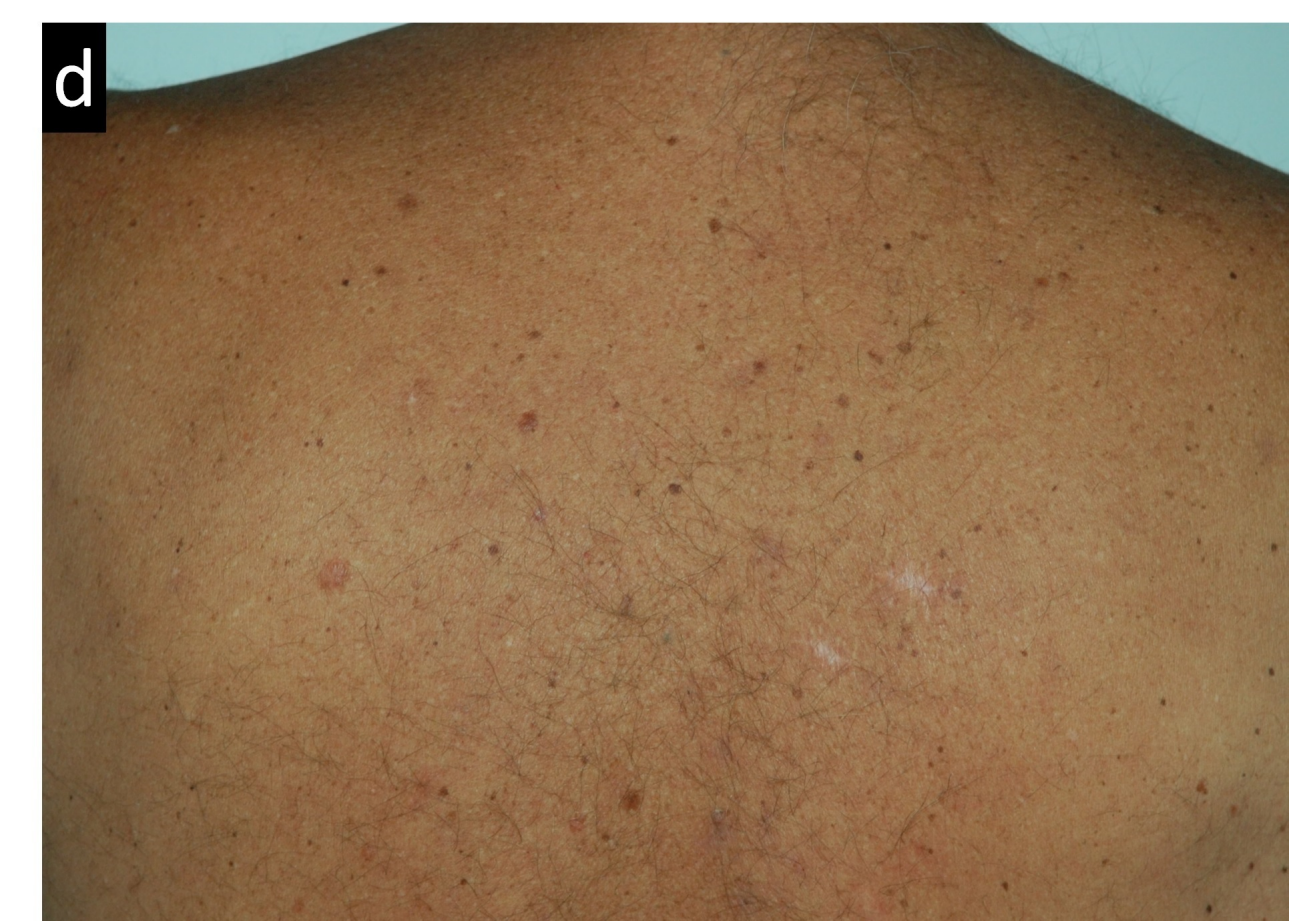
Radiation-induced changes in the patient’s back appearance 7.5 months after radiation therapy

**Figure 7:**
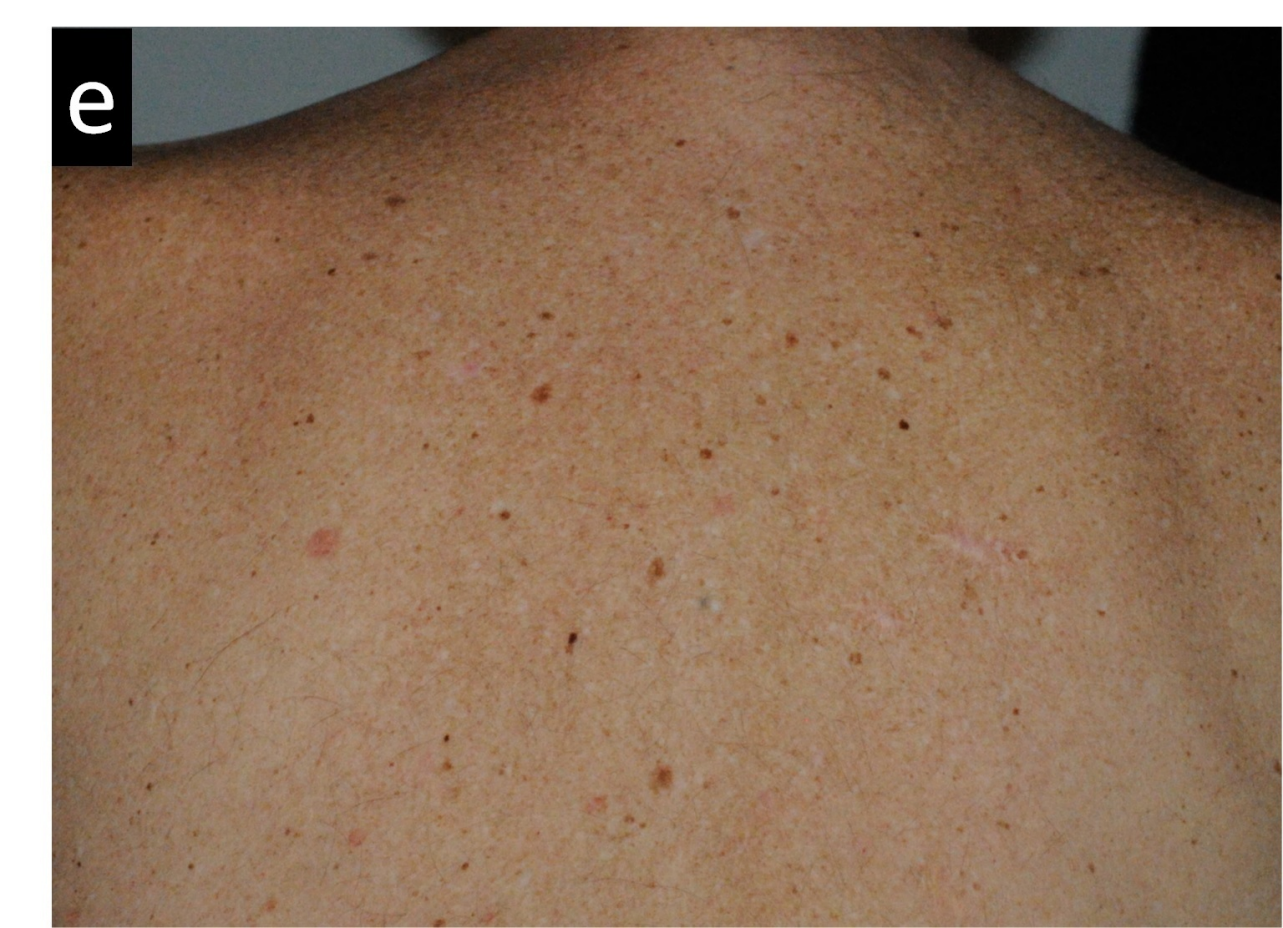
Radiation-induced changes in the patient’s back appearance four years after radiotherapy treatment

Figures 3-7 present images of the patient during and after this treatment. Some erythema is observed at the end of radiation delivery in Figure 4, lesions became less pronounced compared to the start of treatment shown in Figure 3. Complete response is obvious on three-month follow-up (Figure 5) with a restoration of hair after 7.5 months (Figure 6). Four years after tomotherapy treatment of the skin disease remains in complete control (Figure 7).

## Discussion

Over past decades advances in clinic-pathology, immunochemistry, molecular biology, cytogenetics have led to better understanding and classification of lymphomas [1]. Cutaneous involvement generally occurs as multiple lesions either as primary or secondary to systemic disease with variable behavior and outcome at any age, sex, ethnicity [2-4]. Primary cutaneous lymphomas are well reported [2-3], but only a few publications on secondary types are available [4]. One large series from Korea of 106 patients with secondary cutaneous involvement [10] showed 29% of diffuse large bone marrow cells (B-cell) variety, 21% of peripheral thymus cells (T-cells), 19% of natural killer T cells (NK/T-cells), 10% anaplastic, 7% lymphoblastic, 5% angioimmunoblastic, and others. Though frequencies vary among countries, improved survival is expected due to modern chemotherapies, including transplant.

Our case is unique as a long follow-up of 24 years of previously irradiated follicular B-cell limited stage lymphoma with subsequent multiple relapses including skin. The skin lesions started overhead initially, then back of chest as maculo-nodular of follicular-diffuse variety with CD10, CD20, Bcl-6, Bcl-2 positive. These lesions of varying size were more modular on scalp than chest and had developed soon after cyclophosphamide, doxorubicin, vincristine, and prednisone+rituximab (CHOP+R) chemotherapy for transformed lymphoma of the oral cavity. The location and appearance in our patient were similar to the literature on B-cell lymphoma. Photodynamic therapy had limited response. In the case of symptomatic painful lesions and for cosmetic reasons, palliative radiotherapy is often considered as the standard of care using electron beam therapy [5,7]. Complete response (CR) rate of 50% was reported [7], but we believe that generous target volume and higher CR are needed for future transplant and chemotherapy. Although lymphomas are particularly radiosensitive, the relative radiosensitivity of various types of precursor B- or T-cell malignant cells is not clearly defined based on tumor burden, cell populations, probably some transformed chemorefractory cells leading to a wide range of individual cells radiosensitivity. Currently, a standard dose of 24 to 36 Gy in 1.8 to 2 Gy per fraction is usual prescription. Helical tomotherapy delivered 30 Gy in 15 fractions to a fairly large volume of the chest and posterior shoulders as shown in Figure 1 instead of multiple fields of electron beam therapy using tomotherapy advantages of image guidance, dose homogeneity, and better dose distribution in the planning target volume (PTV). The PTV included nodules in deep dermis seen on CT with 0.5 cm isotropic margin to account for patient motion in the prone position. Except for moderate erythema and pigmentation, there were no acute or delayed toxicities, over two months there was a complete response. The scalp lesion responded similarly, however, 1.5 years later a progressive relapse in lymph L-nodes was detected. The patient received conditioning chemo plus stem cell transplant with no compromise due to previous tomotherapy treatment. The patient remains presently well in remission four years since skin treatment on tomotherapy.

## Conclusions

Treatment of B-cell type macular-nodular lesions over a large volume of back and posterior shoulders on a helical tomotherapy radiotherapy system is reported. The skin lesions responded completely with no toxicity. Palliative radiotherapy to a fairly large and complex volume of skin with modest dose avoiding underlying critical tissues on tomotherapy is feasible, well tolerated with excellent durable response during four years follow-up, without compromising future chemotherapy and stem cell transplant for systemic relapse.
